# Decrease in drug accumulation and in tumour aggressiveness marker expression in a fenretinide-induced resistant ovarianumour cell line

**DOI:** 10.1054/bjoc.2001.1826

**Published:** 2001-06

**Authors:** V Appierto, E Cavadini, R Pergolizzi, L Cleris, R Lotan, S Canevari, F Formelli

**Affiliations:** Department of Experimental Oncology, Istituto Nazionale Tumori, 20133 Milan, Italy; Department of Thoracic and Head and Neck Medical Oncology, MD Anderson Cancer Center, 77030 Houston, Texas, USA

**Keywords:** retinoids, ovarian tumour, fenretinide-resistance, drug uptake, differentiation, RARβ

## Abstract

We investigated whether the efficacy of fenretinide (HPR) against ovarian tumours may be limited by induction of resistance. The human ovarian carcinoma cell line A2780, which is sensitive to a pharmacologically achievable HPR concentration (IC _50_= 1 μM), became 10-fold more resistant after exposure to increasing HPR concentrations. The cells (A2780/HPR) did not show cross-resistance to the synthetic retinoid 6-[3-adamantyl-4-hydroxyphenyl]-2-naphthalene carboxylic acid (CD437) and were not sensitive, similarly to the parent line, to all- *trans* -retinoic acid, 13- *cis* -retinoic acid or N-(4-methoxyphenyl)retinamide. A2780/HPR cells showed, compared to parental cells, a 3-fold reduction in colony-forming ability in agar. The development of HPR resistance was associated with a marked increase in retinoic acid receptor β (RARβ) mRNA and protein levels, which decreased, together with drug resistance, after drug removal. The expression of cell surface molecules associated with tumour progression including HER-2, laminin receptor and β1 integrin was markedly reduced. The increase in the levels of reactive oxygen species is not involved in HPR-resistance because it was similar in parental and resistant cells. Conversely differences in pharmacokinetics may account for resistance because, in A2780/HPR cells, intracellular peak drug levels were 2 times lower than in A2780 cells and an as yet unidentified polar metabolite was present. These data suggest that acquired resistance to HPR is associated with changes in marker expression, suggestive of a more differentiated status and may be explained, at least in part, by reduced drug accumulation and increased metabolism. © 2001 Cancer Research Campaign http://www.bjcancer.com


*British Journal of Cancer* (2001) **84**(11), 1528–1534
© 2001 Cancer Research Campaign
doi: 10.1054/ bjoc.2001.1826, available online at http://www.idealibrary.com on IDEAL®

http://www.bjcancer.com

### Decrease in drug accumulation and in tumour aggressiveness marker expression in a fenretinide-induced resistant ovarian tumour cell line

**V Appierto[1], E Cavadini[1], R Pergolizzi[1], L Cleris[1], R Lotan[2], S Canevari[1] and F Formelli[1]**

[1]Department of Experimental Oncology, Istituto Nazionale Tumori, 20133 Milan, Italy; [2]Department of Thoracic and Head and Neck Medical Oncology, MD Anderson Cancer Center, 77030 Houston, Texas, USA

**Summary** We investigated whether the efficacy of fenretinide (HPR) against ovarian tumours may be limited by induction of resistance. The human ovarian carcinoma cell line A2780, which is sensitive to a pharmacologically achievable HPR concentration ($IC_{50}$ = 1 μM), became 10-fold more resistant after exposure to increasing HPR concentrations. The cells (A2780/HPR) did not show cross-resistance to the synthetic retinoid 6-[3-adamantyl-4-hydroxyphenyl]-2-naphthalene carboxylic acid (CD437) and were not sensitive, similarly to the parent line, to all-*trans*-retinoic acid, 13-*cis*-retinoic acid or N-(4-methoxyphenyl)retinamide. A2780/HPR cells showed, compared to parental cells, a 3-fold reduction in colony-forming ability in agar. The development of HPR resistance was associated with a marked increase in retinoic acid receptor β (RARβ) mRNA and protein levels, which decreased, together with drug resistance, after drug removal. The expression of cell surface molecules associated with tumour progression including HER-2, laminin receptor and β1 integrin was markedly reduced. The increase in the levels of reactive oxygen species is not involved in HPR-resistance because it was similar in parental and resistant cells. Conversely differences in pharmacokinetics may account for resistance because, in A2780/HPR cells, intracellular peak drug levels were 2 times lower than in A2780 cells and an as yet unidentified polar metabolite was present. These data suggest that acquired resistance to HPR is associated with changes in marker expression, suggestive of a more differentiated status and may be explained, at least in part, by reduced drug accumulation and increased metabolism. © 2001 Cancer Research Campaign http://www.bjcancer.com

**Keywords**: retinoids; ovarian tumour; fenretinide-resistance; drug uptake; differentiation; RARβ

N-(4-hydroxyphenyl)retinamide (HPR) is a synthetic retinoic acid analogue under investigation in clinical trials as preventive and therapeutic agent. In animal models HPR has shown chemopreventive efficacy against mammary gland, urinary bladder, seminal vesicle and prostate carcinogenesis and therapeutic efficacy against mammary, prostate and ovarian tumours (Formelli et al, 1996). In vitro studies have demonstrated that HPR has significant antiproliferative activity associated with induction of apoptosis in several tumour cell types including breast, prostate, leukaemia, head and neck, neuroblastoma and ovary (Formelli et al, 1996). The mechanism of action of HPR has not yet been clarified. Some studies suggest that the effects of this retinoid are mediated through signalling of retinoic acid receptors (RARs) and retinoid X receptors (RXRs) (Fanjul et al, 1996; Supino et al, 1996; Pergolizzi et al, 1999). Other results suggest that HPR can act as a pro-oxidant and induce apoptosis by eliciting oxidative stress (Oridate et al, 1997). In humans, HPR has already shown efficacy in oral leukoplakia and lichen planus (Formelli et al, 1996). Recently, reductions in the incidence of contralateral breast cancer and ipsilateral breast cancer reappearance have been reported in premenopausal women with early breast cancer treated with HPR (Veronesi et al, 1999). Promising preliminary results suggest that HPR can protect against ovarian cancer (De Palo et al, 1995; Veronesi et al, 1999) and clinical trials are in progress to test the effects of the retinoid against the tumour.

A characteristic of retinoid treatment is that discontinuation of treatment leads to recurrence of the lesion. However, as occurs with chemotherapeutic drugs, continuous retinoid treatment might cause development of drug resistance. Since no data are available on resistance to HPR, we investigated whether this event occurs with the retinoid and attempted to evaluate the molecular basis of the effect. HPR-resistant cells (A2780/HPR), were developed by continuous in vitro exposure to HPR of A2780 human ovarian carcinoma cells, which have been shown to be very sensitive to the growth-inhibitory effect of HPR (Supino et al, 1996). To understand the possible basis of the decreased HPR sensitivity, we investigated whether the generation of reactive oxygen species and HPR uptake/metabolism are altered in HPR-resistant cells. The pattern of RAR expression and the expression of markers associated with differentiation and tumour progression was also examined and compared with that of sensitive cells.

### MATERIALS AND METHODS

### Cell culture, conditions and drugs

HPR resistant cells were obtained by culturing parental A2780 cells with increasing concentrations of HPR. Cells were initially cultured with 1 μM HPR and drug concentration was increased by 1 μM every 5 passages up to 5 μM. Then cells were maintained

*Received 6 July 2000*
*Revised 18 January 2001*
*Accepted 1 February 2001*

*Correspondence to:* F Formelli continuously in 5 μM HPR (A2780/HPR). The cells were grown in monolayer in RPMI 1640 medium containing 10% fetal bovine serum (FBS) in 5% $CO_2$ at 37˚C. HPR, all-*trans*-retinoic acid (RA), 13-*cis*-retinoic acid (13-*cis*-RA), N-(4-methoxyphenyl)retinamide (MPR), and 6-[3-adamantyl-4-hydroxyphenyl]-2-naphthalene carboxylic acid (CD437) were dissolved at 30 mM in DMSO prior to further dilution in culture medium. HPR and MPR were kindly provided by RW Johnson Pharmaceutical Research Institute (Spring House, PA, USA) and CD437 by CIRD/Galderma (Sophia Antipolis, France). RA and 13-*cis*-RA were purchased from Sigma (Milan, Italy).

Cell diameters were measured by Coulter Counter Multisizer II (Coulter, Luton, UK).

### Growth inhibition assay

Growth inhibition assays were performed as previously described (Supino et al, 1996). Briefly, cells were treated with different concentrations of the drugs, 24 h after seeding, for 3 days. Cell number was determined by counting trypsinized cells using a ZBI electronic particle Counter (Coulter). All data are presented as means of duplicate cultures obtained from at least 2 independent experiments.

### Colony formation assay

For determination of the clonogenic ability, 35-mm diameter dishes were first layered with 0.5% agarose/2× RPMI supplemented with 10% FBS. A top layer containing 5000 cells plate$^{-1}$ suspended in 0.3% agarose/2× RPMI supplemented with 10% FBS was then added. For the assay HPR was removed from the medium of the resistant cells. Dishes were maintained in 5% $CO_2$ at 37˚C for 9 days.

### Northern analysis

Total cellular RNA was isolated by the guanidium thiocyanate-CsCl method. About 20 μg of total RNA was fractionated on a 1% agarose gel, transferred to nylon filters and probed with $^{32}$P-labelled hRARα, hRARβ or hRARγ cDNAs, as previously reported (Supino et al, 1996). A similarly labelled human glyceraldehyde-3-phosphate dehydrogenase (GAPDH) cDNA probe was used for assessment of RNA loading.

### Immunoblot analysis

Cells were lysed in Laemmli sample buffer containing 5% β-mercaptoethanol and boiled for 3 minutes. Aliquots containing 80 μg of total cell proteins were fractionated on 12% SDS-PAGE and transferred to nitrocellulose membranes (Amersham Life Science Inc, Arlington Heights, IL, USA). Membranes were blocked in 5% non-fat milk in Tris-buffered saline (TBS) for 1 h at room temperature and then incubated overnight at 4˚C with rabbit polyclonal antibodies raised against hRARβ (Santa Cruz Biotechnology Inc, Santa Cruz, CA, USA). After washing in TBS containing 0.1% tween 20, the filters were incubated with peroxidase antirabbit immunoglobulin G and specific complexes were revealed by chemiluminescence according to the ECL (Enhanced Chemiluminescence) Western blotting detection system Kit (Amersham).

### Flow cytometric analysis

Cells, either living or after cell fixation/permeabilization with 70% cold ethanol for 20 min, were incubated with primary antibody (10 μg ml$^{-1}$ in phosphate-buffered saline containing 0.03% bovine serum albumin) for 30 min on ice. Primary monoclonal antibodies purified from hybridomas maintained in the laboratory (Dolo et al, 1997) were: MAR4 against β1 integrin, W6/32 against HLA class I (ATCC, Rockville, MD), MluC5, against the 67 kDa laminin receptor (LNR), Mint5 against the HER-1/EGFR (HER-1), MGR6, against HER-2/ErbB-2 (HER-2), Mov18, directed against the folate receptor α (αFR). Commercial monoclonal antibodies or rabbit polyclonal antibodies used were: anti-caveolin 1, purchased from Santa Cruz (Santa Cruz Biotechnologies) and anti-cytokeratins 8/18 purchased from Novo Castra (Novo Castra Laboratories Ltd, Newcastle-upon-Tyne, UK). After washing with PBS, cells were incubated with fluorescein-labelled goat anti-mouse IgG (Kirkegaard and Perry Laboratories Inc, Gaithersburg, MD, USA) for 30 min on ice. Cell-associated fluorescence was analysed using a flow cytometer (FACScan, Becton Dickinson, Collaborative Research, Bedford, MA, USA). Expression levels for each marker were evaluated as fluorescence intensity in the presence of the relevant antibody and compared to the background-staining value obtained from cells incubated with fluoresceinated antibody only.

### Tumorigenicity assay

Cultured cells were harvested by trypsinization, washed and resuspended in 0.9% NaCl. Cell suspensions were injected s.c. as $2.5 \times 10^6$ cells and i.p. as $15 \times 10^6$ cells which correspond to the oncogenic doses 100% s.c. and i.p. respectively of A2780 cells. Cells were injected in 0.2 ml volume in 7–9-week-old CD-1 nu/nu female mice (Charles River, Calco, Italy). Mice were monitored for tumour onset and tumour growth twice a week. In s.c.-injected mice, tumour diameters were measured by a caliper, and tumour weights were estimated according to the formula: weight (g) = [width (cm)]$^2$ × length (cm)/2. 2 experiments were performed, and the growth of the resistant cells was compared with that of the parental cells. Statistical analysis was performed by Fisher's exact test for evaluation of tumour takes (i.e., number of tumours developed out of number of tumour injections) and the 2-tailed Student's *t*-test for the evaluation of tumour growth (i.e., number of days to reach a tumour weight of 0.1 and 1 g).

Animal studies were reviewed and approved by the ethics Committee for Animal Experimentation of the Istituto Nazionale Tumori (Milan, Italy) and are in accordance with the guidelines of the UK Coordinating Committee on Cancer Research (UKCCCR, 1988).

### Measurement of intracellular reactive oxygen species

The intracellular generation of reactive oxygen species was measured by use of the oxidation-sensitive fluorescent dye 2′, 7′-dichlorofluorescein diacetate (DCF-DA) (Molecular Probes, Eugene, OR, USA). Cells were seeded at $1 \times 10^5$ cells well$^{-1}$ in 48 wells plates. 24 hours after seeding, cells were washed twice with Hank's balanced salt solution (Gibco BRL, Gaithersburg, MD, USA) and 250 μl Hank's balanced salt solution were added. HPR was added at a final concentration of 10 μM and DCF-DA at a final concentration of 10 μg ml$^{-1}$. After 15 minutes incubation at

© 2001 Cancer Research Campaign 

37˚C, the fluorescence was measured at 15 minutes intervals for up to 190 minutes at 530 nm after excitation at 480 nm in a cytofluorometer 2350 Fluorescence Measurement System (Millipore Bedford, MA, USA).

### HPR uptake and metabolism

The intracellular content of HPR was evaluated by HPLC as previously described (Formelli et al, 1993; Supino et al, 1996). Cells were seeded and incubated for 24 h. They were then treated with 1 µM HPR and 1, 4, 6, 8, 24, 48 and 72 h after HPR addition, medium was removed and cells were scraped and washed 3 times in 0.9% NaCl. The pellets were then frozen and kept for up to 1 week at –20˚C until drug assay. After thawing, cell pellets were resuspended in 1 ml of distilled water containing 125 µg $ml^{-1}$ of butylated hydroxytoluene (BHT) (Sigma, St Louis, MO, USA) as anti-oxidant and sonicated. 200 µL of each cell sample were added to 400 µl $CH_3CN$ containing 125 µg $ml^{-1}$ BHT, vortex mixed and centrifuged to pellet the precipitated proteins. The recovered supernatants were analysed on a liquid chromatograph (Perkin Elmer, Norwall, CT, USA) fitted with a C18 (5 µm) reverse-phase column (150 × 4.6 mm) and a C18 precolumn (Perkin Elmer, Milan, Italy). The mobile phase consisted of $CH_3CN$:$H_2O$:$CH_3COOH$ (75:23:2, vol/vol/vol) delivered at a flow rate of 2 ml $min^{-1}$. Detection was performed with a Perkin Elmer LC95 absorbance detector at 340 nm. N-(4-etoxyphenyl)-retinamide (EPR) was used as internal standard by adding it to the $CH_3CN$ used to precipitate the proteins. The reference standards HPR, MPR and the internal standard EPR were supplied by the RW Johnson Pharmaceutical Research Institute. RA and retinol, used as reference standard were obtained from Sigma. All the procedures were performed with the samples protected from light. The protein content of each sample was evaluated in parallel by the BCA Protein Assay Reagent (Pierce, Rockford, IL, USA).

### RESULTS

### A2780/HPR cells: morphological and growth characteristics and sensitivity to other retinoids

HPR-resistant cells were obtained after 20 passages of A2780 cells by culturing them with increasing concentration of HPR up to 5 µM. These cells were subsequently maintained in the presence of the highest employed concentration. HPR resistance was not associated with changes in the proliferation rate (doubling time = 19–20 h). The effect of HPR on the proliferation of parental and HPR-resistant cells was evaluated after 3 days of exposure to the drug (Figure 1). In A2780 cells 50% growth inhibition was achieved with approximately 1 µM HPR. The resistant index (RI) of resistant cells was slightly higher than 10. To assess the stability of HPR resistance, A2780/HPR cells were split and subcultured for 5 passages without HPR (A2780/HPR/rev) (Figure 1). A2780/HPR/rev cells showed a lower degree of resistance (RI = 5) than A2780/HPR cells. To investigate whether the resistance to HPR was associated to resistance to other retinoids, the effects of RA, 13cis-RA, MPR, and CD437 were evaluated on A2780 and A2780/HPR cells (Figure 1). No differences in sensitivity to CD437 were observed between A2780 and A2780/HPR cells. In both cell lines CD437 caused a strong dose-dependent growth inhibition: 50% growth inhibition was achieved with 0.5 µM. RA and MPR were ineffective in both cell lines at doses up to 10 µM.

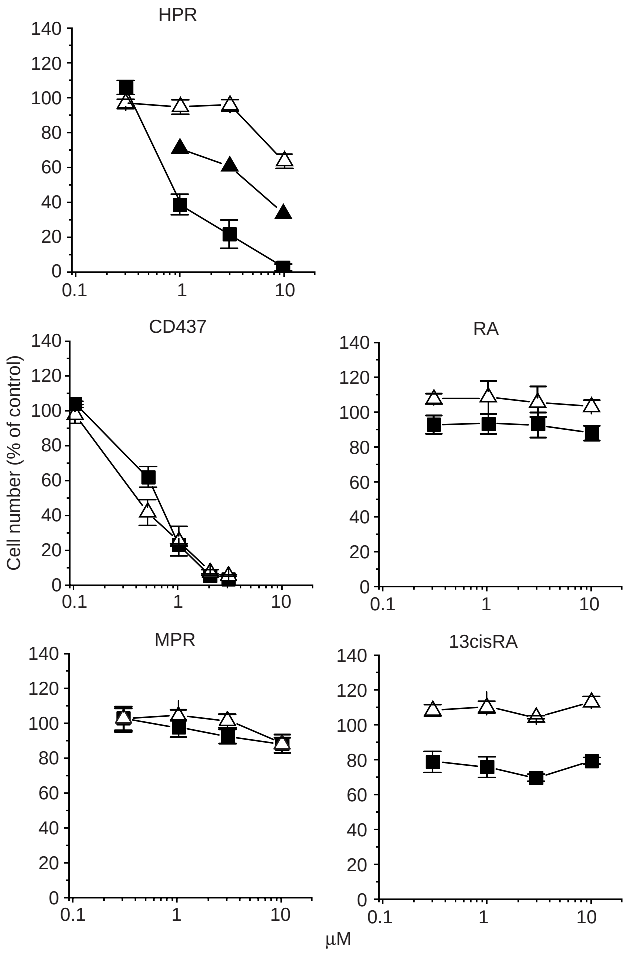

**Figure 1** Antiproliferative effect of HPR, CD437, RA, MPR, and 13-*cis*-RA on A2780 (■) and A2780/HPR (Δ) cells. The effect of HPR was also evaluated on A2780/HPR/rev (▲) cells. Cells were treated 24 h after seeding and surviving cell number was evaluated 3 days later. Data are the means of 3 independent experiments

Relative to the parental counterpart, A2780/HPR cells showed limited cross-resistance to 13cis-RA. The retinoid caused a slight dose-independent growth inhibition of A2780 cells whereas it was ineffective in A2780/HPR cells.

Morphological differences between parental and resistant cells could be observed (data not shown). A2780 parental cells were heterogeneous in shape. In contrast resistant cells were round, and reduced in size. The average cell diameter, evaluated in the stationary phase, was 12 µm for resistant cells and 15 µm for parental cells. Additionally, resistant cells formed foci-like aggregates.

The clonogenic assay, performed in agar, showed a 3-fold reduction in colony-forming ability of A2780/HPR cells compared with parental cells (plating efficiency 3.52 ± 0.063% vs 1.05 ± 0.4%) when a cut-point of 100 µm for colony diameter was fixed. The reduction in colony-formation ability of resistant cells was associated with a 3-fold reduction in mean colony diameter (584 ± 24 µm vs 146 ± 3.46 µm) (data not shown). The observed reduction in colony formation ability of A2780/HPR cells was not due to possible exclusion of some colonies because of their smaller size. In fact a reduction was also observed by counting colonies of more than 50 cells (plating efficiency 5.15 ± 0.052% vs 2.11 ± 0.21%).

 *© 2001 Cancer Research Campaign*

### RARs expression in A2780/HPR cells

To understand whether nuclear RARs are involved in the development of HPR resistance, RAR α, β and γ mRNA levels were evaluated in parental and resistant cells. A2780/HPR cells showed RARα and RARγ mRNAs levels similar to those of A2780 cells (data not shown). However RARβ expression in resistant cells was markedly increased at the mRNA and protein level (Figure 2A and 2B, respectively). In A2780/HPR/rev cells, obtained by culturing resistant cells without the drug for 5 passages (Figure 2B), RARβ protein expression was decreased compared to A2780/HPR cells, but the levels were still higher than those of the parental line.

### Effect of HPR resistance on expression of markers of adhesion, differentiation and tumour progression

FACs analysis (Table 1) revealed no significant difference between A2780 and A2780/HPR cells in expression of HLA class I molecules on the cell surface and vimentin within the cells. Moreover caveolin 1 expression, which characterizes the internal leaflet of the plasma membrane of endothelia, fibroblasts and several differentiated epithelia, was not modified upon acquisition of HPR resistance. In contrast cytokeratin 8/18 was reduced and the level of expression of β1 integrin, which was already low, dropped below the limit of detectability in resistant cells. Among the analysed tumour progression markers, the negative expression of HER-1 and α-FR, a marker of non-mucinous ovarian carcinoma, in parental cells was unaffected. Conversely LNR was reduced and the level of expression of HER-2 was below the limit of detectability in the resistant line A278/HPR.

### Tumorigenicity of A2780/HPR cells

To study the possible role of the increased expression of RARβ, the reduced colony-forming ability and the reduced expression of markers associated with aggressiveness in the in vivo tumorigenicity of cells made resistant to HPR, A2780/HPR cells were injected s.c. or i.p. into nude mice and their growth was compared with that of the parental line (Table 2). The number of takes and the growth pattern of A2780/HPR cells did not differ from that of the parent line after s.c. or i.p. injection.

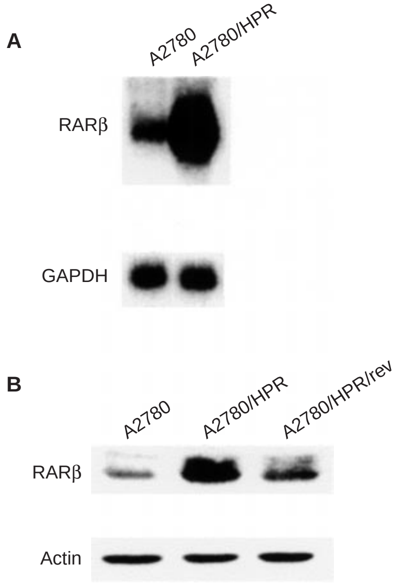

**Figure 2** (**A**) Northern analysis for RARβ expression in A2780 and A2780/HPR cells. As a control for loading, the filters were stripped and rehybridized with GAPDH. (**B**) Western analysis for RARβ expression in A2780, A2780/HPR and A2780/HPR/rev cells. As a control for loading the blots were incubated with β-actin antibody

### Generation of reactive oxygen species in A2780 and A2780/HPR cells

The effect of HPR on generation of reactive oxygen species was measured in A2780 and A2780/HPR cells. Cells were treated with a high HPR concentration (10 μM) in order to better detect reactive oxygen species generation at short times after drug exposure. No difference in the rate or levels (250% increase compared to controls at 180 min) of reactive oxygen species was found between sensitive and resistant cells (data not shown).

### HPR uptake and metabolism in A2780 and A2780/HPR cells

The intracellular drug content of A2780 and A2780/HPR cells was evaluated by HPLC analysis. Cells were exposed for 72 h to 1 μM HPR, a concentration which corresponds to the $IC_{50}$ in A2780 cells and which is inactive in A2780/HPR cells. The representative chromatograms of cells after 72 h in the presence of HPR, are presented in Figure 3A. The initial peak (a), coeluting with the front of the solvent, and peak b, corresponding to BHT, used as antioxidant, were also found in cells not exposed to HPR. In cell extracts from A2780 cells, only 2 peaks were found: peak 2 (retention time (R.T.) = 3.3 min), corresponding to an unidentified metabolite, and peak 3 (R.T. = 3.7 min), corresponding to HPR. In A2780/HPR cells besides peaks 2 and 3, an extra peak with a R.T. of 1.3 min was found. No peak corresponding to RA (R.T. = 4.2 min), to retinol (R.T. = 4.6 min) or to MPR (R.T. = 6.3 min) was detected in A2780 and A2780/HPR cells. The levels of HPR and of the unidentified metabolites, as ng $mg^{-1}$ proteins, in sensitive and in resistant cells are reported in Figure 3B. If the levels are expressed as ng $cell^{-1}$ number, the results are comparable because sensitive and resistant cells had similar protein levels. The time course study showed that A2780 and A2780/HPR cells both reached peaks of intracellular HPR concentrations at 4 h. The peak levels of HPR in A2780/HPR cells were 2 times lower than those of the parent line (1280 vs 2496 ng $mg^{-1}$ protein). Nonetheless, drug levels at later observation times (48–72 h) were similar and the areas under the curves ($AUC_{0-72\,h}$) were 45.9 and 63.1 μg $h^{-1}$ $mg^{-1}$ protein for resistant and sensitive cells, respectively. The unidentified metabolites (peak 2 in A2780 and peaks 1 and 2 in A2780/HPR cells) were detected starting at 24 h. The concentrations of each metabolite, evaluated as HPR equivalents, represented, at 72 h, approximately 20% of the total retinoid content.

### DISCUSSION

In the study we have analysed the mechanisms and the molecular characteristics associated with the induction of HPR resistance in a human ovarian carcinoma cell line. We hypothesized that HPR resistance might occur, as with cytotoxic drugs, due to continuous drug exposure. We have shown that when A2780 ovarian tumour cells, which are very sensitive to the growth inhibitory effect of HPR, were exposed continuously to the retinoid HPR, they

© 2001 Cancer Research Campaign 

**Table 1** Expression of markers on parental ovarian carcinoma cell line A2780 and HPR resistant cell line A2780/HPR[a]

| Marker | A2780 % | A2780 MFI | A2780/HPR % | A2780/HPR MFI | Variation |
|---|---|---|---|---|---|
| HLA class I | 36.3 | 10.42 | 39.4 | 10.96 | ⇔(+) |
| Caveolin 1* | 99.7 | 182.60 | 99.0 | 164.29 | ⇔(+) |
| Vimentin* | 95.4 | 210.58 | 97.6 | 195.86 | ⇔(+) |
| Cytokeratin 8/18* | 55.9 | 33.39 | 33.2 | 22.04 | ⇓(+) |
| β1 integrin | 20.9 | 19.68 | 10.0 | 6.79 | ⇓(+ to –) |
| HER 1 | 7.5 | 8.68 | 6.9 | 9.13 | ⇔(–) |
| αFR | 9 | 18.83 | 9 | 22.02 | ⇔(–) |
| LNR | 33 | 45.03 | 24.5 | 23.13 | ⇓(+) |
| HER 2 | 40.1 | 18.53 | 19.6 | 6.68 | ⇓⇓ (+ to –) |

(Marker expression)

[a]By FACS analysis on live or permeabilized cell suspensions: the reported data refer to a single experiment. Similar data were obtained in 2–4 repeated experiments. % = % of positive cells. MFI = mean fluorescence intensity of positive cells. Cut off level of positivity (+): more than 10% with a MFI > 10%. Negative staining (–) i.e. no specific fluorescence over the background level in presence of the FITC fluorescein-labelled secondary antibody alone. ⇔ = reduction of total fluorescence (% of positive cells × MFI) < 20%; ⇓ = reduction 20–80%; ⇓⇓ = reduction > 80%.
*Cells were permeabilized with 70% EtOH on ice for 20 minutes.

became resistant. The growth of A2780 cells was 50% inhibited by 1 μM HPR, a pharmacologically achievable concentration (Formelli et al, 1993), whereas concentrations 10 times higher, probably associated with severe side effects, were required to obtain the same effects in A2780/HPR cells. The resistance to HPR was reversible, thus indicating that the presence of the drug is necessary to maintain the resistance phenotype. Resistance to HPR was not associated to differences in sensitivity to the synthetic retinoid CD437. Consistent with this finding, it was recently reported that an acute promyelocytic leukaemia cell line, made resistant to CD437, did not display any cross-resistance to HPR (Ponzanelli et al, 2000). Such results indicate that the 2 retinoids exert their growth inhibitory effects through different mechanisms of action and moreover they suggest that combination of the 2 retinoids might result in improved efficacy. RA and MPR were ineffective against A2780 and A2780/HPR cells whereas 13-cisRA showed a slight level of cross-resistance with 4-HPR on A2780/HPR cells. The significance of this effect is not known.

The nuclear retinoid pathway (Fanjul et al, 1996) and oxidative stress (Oridate et al, 1997) have been found to be operative in mediating HPR growth-inhibitory effects. We previously showed that RARs' expression is involved in the apoptotic effect of HPR in parental A2780 cells (Pergolizzi et al, 1999). In the cells, elevation of RARβ expression levels by stable transfection resulted in increased sensitivity to some HPR concentrations. In contrast, in this study, the observed RARβ elevation was associated with HPR resistance. We also previously demonstrated that in A2780 cells, a short (6 h) HPR treatment caused an increase in RARβ mRNA levels (Pergolizzi et al, 1999). Therefore, the marked increase in RARβ expression found in HPR-resistant cells, compared with parental cells, seems to be the result of continuous induction, due to the presence of HPR in the medium, rather than an intrinsic characteristic of resistant cells. The hypothesis is compatible with the fact that when HPR was eliminated from the medium for 5 passages, the levels of RARβ expression decreased. A recent report showed that HPR may exert 2 separate effects in F9 teratocarcinoma cells: early cell killing at high doses (10 μM) and late differentiation at low doses (1 μM) (Clifford et al, 1999). Likewise in our system, cells surviving HPR continuous treatment showed a more differentiated morphology than parental cells. The expression of some of the investigated markers was compatible with enhanced differentiation. Parental A2780 cells coexpressed vimentin, which is a component of stromal matrix origin, and cytokeratin 8/18 which is characteristic of simple epithelia. The observed reduction of cytokeratin 8/18 might be a consequence of the induction of a more differentiated phenotype. The expression of HER-2, a transmembrane glycoprotein receptor which is overexpressed in approximately 30% of ovarian carcinoma, and which

**Table 2** Tumorigenicity of parental ovarian carcinoma cell line A2780 and HPR-resistant cell line A2780/HPR[a]

| Cells | s.c. injection: No. of tumours/ No. of injections | s.c. injection: Tumour latency (days at 0.1 g) | s.c. injection: Tumour growth (days at 1 g) | i.p. injection: No. of tumours/ no. of injections | i.p. injection: Tumour latency (days at tumour onset) |
|---|---|---|---|---|---|
| A2780 | 8/9 | 10.9 ± 3.9 | 16.7 ± 7.2 | 9/10 | 31.2 ± 7.6 |
| A2780/HPR | 8/8 | 7.7 ± 2.2 | 16.2 ± 3.6 | 8/10 | 30.4 ± 5.1 |

[a]Mice were injected s.c. with 2.5 × 10⁶ cells and i.p. with 15 × 10⁶ cells which correspond to the oncogenic doses 100% of A2780 cells after i.p. and s.c. route, respectively. Mice were checked for tumour latency and growth (mean ± SD) as described in Material and Methods.

 © 2001 Cancer Research Campaign

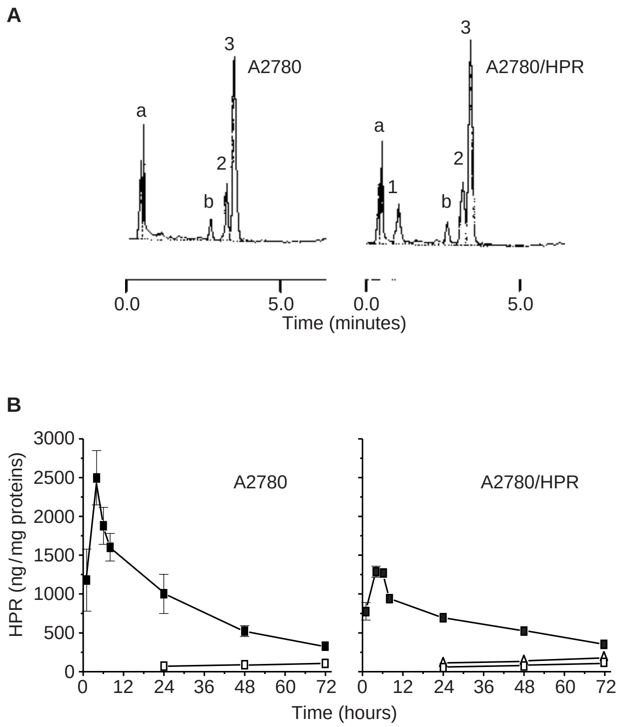

**Figure 3** (**A**) Chromatograms of A2780 and A2780/HPR cells exposed to 1 μM HPR for 72 h. Only the first 6.5 minutes of the chromatograms have been reported, because no peak besides the internal standard EPR (R.T. = 7.9 min) was found from 6.5 to 10 minutes. a: peak found also in not treated cells; b: BHT, used as antioxidant; 1: unidentified metabolite (R.T. = 1.3 min); 2: unidentified metabolite (R.T. = 3.3 min); 3: HPR (R.T. = 3.7 min). (**B**) HPR uptake in A2780 and A2780/HPR cells. Cells were incubated with 1 μM HPR. Drug uptake was evaluated at different times up to 72 h. Results are expressed as average ± SD of one experiment done in duplicate representative of three experiments. HPR (■); peak 1 expressed as HPR equivalent (Δ); peak 2 expressed as HPR equivalent (□)

correlates with poor survival (Berchuck et al, 1990) dropped below the level of detectability. Laminin receptor expression, which is correlated with ovarian tumour progression (Van der Brule et al, 1996) was decreased. Finally β1 integrin, which has a relevant role in inflammation, cell adhesion and migration and which is activated during tumour progression and invasion, disappeared, thus suggesting a reduced metastatic phenotype of resistant cells. The disappearance of β1 integrin from the cell surface and the reduced levels of LNR are also consistent with the change in morphology of A2780 HPR resistant cells. Reduction in HER-2 (Pellegrini et al, 1995) and β1 integrin (Rozzo et al, 1997) expression have also been found in other tumour cells after a single HPR treatment. The continuous expression of RARβ, a putative tumour suppressor (Houle et al, 1993) and the reduced expression of tumour aggressiveness markers may be the reason for the lower colony-forming potential of the HPR-resistant cells. However, the reduced clonogenic ability of resistant cells was not sufficient to result in reduced in vivo tumorigenicity.

Our results indicate that HPR-resistance is not related to different rates of generation of intracellular reactive oxygen species and that reactive oxygen species are not important mediators of HPR-induced growth inhibitory effects in A2780 cell.

As it often occurs with cytotoxic drugs, resistance induced by continuous exposure to HPR was associated with decreased intracellular drug levels. The peak levels of HPR were 2 times lower in resistant cells and the $AUC_{0-72\,h}$ was reduced by 30%. The mechanism responsible of the cellular HPR uptake is not known. The reduced peak levels might be due to impairment in drug uptake or they might reflect the reduced volume of resistant cells. At variance with peak levels, the intracellular HPR levels at late observation times (48–72 h) did not differ in the 2 cell types, thus indicating similar late drug release. Interestingly, in NB4 leukaemia cells, made resistant to the synthetic retinoid CD437, resistance was not associated to decreased drug uptake (Ponzanelli et al, 2000).

Besides showing reduced peak levels, resistant cells showed a different pattern of HPR metabolism. A polar metabolite, not detected in sensitive cells, was found in cell extracts from resistant cells. Perhaps cells sensitive to HPR activate, after continuous exposure to the drug, new catabolic pathways in order to protect themselves from the drug growth inhibitory effects. It is interesting to note that in tumour cells, sensitive and naturally resistant to RA, sensisitivity to the growth inhibitory effect of the retinoid was correlated with the ability to metabolize it to polar metabolites (Braakhuis et al, 1997; Van der Leede et al, 1997). The nature and the role of the polar metabolite found in resistant cells are currently being elucidated in our laboratory. Another metabolite slightly more polar than HPR was found in sensitive and resistant cells. This compound is not an impurity in HPR because it was not found in the administered HPR solution and its concentrations increased with time. Neither RA nor MPR, a major HPR metabolite, which reaches in human plasma concentrations similar to those of the parent drug (Formelli et al, 1993), was detected in either cell line, thus indicating that such metabolic pathways do not occur in the cells.

Up to now only few in vitro models had been developed to study mechanisms and characteristics of acquired retinoid resistance. Here we have described the first in vitro model of acquisition of HPR resistance. In summary, we have presented evidence that continuous exposure of tumour cells to HPR results in induction of HPR resistance. HPR resistance is associated with decreased peak drug levels and with changes in marker expression that may be interpreted as indicative of increased cell differentiation. We have also shown that HPR metabolism in A2780/HPR cells is different than in parental cells. Should this metabolic pathway occur in all HPR resistant cells, it might represent a marker for HPR-induced resistance.

### ACKNOWLEDGEMENTS

The study was supported by grants from the Associazione Italiana per la Ricerca sul Cancro, Milan, and from the Consiglio Nazionale delle Ricerche, Rome, Italy. We thank Ms L Zanesi for manuscript preparation.

### REFERENCES

Berchuck A, Kamel A, Whitaker R, Kerns B, Olt G, Kinney R, Sopert JT, Dodge R, Clarke-Pearson DL, Marks P, Mckenzie S, Yin S and Bast RC Jr (1990) Overexpression of HER-2/*neu* is associated with poor survival in advanced epithelial ovarian cancer. *Cancer Res* **50**: 4087–4091

Braakhuis BJM, Klaassen I, Van Der Leede BM, Cloos J, Brakenhoff RH, Copper MP, Teerlink T, Hendriks HFJ, Van Der Saag PT and Snow GB (1997) Retinoid metabolism and all-trans retinoic acid-induced growth inhibition in head and neck squamous cell carcinoma cell lines. *Br J Cancer* **76**: 189–197

Clifford JL, Menter DG, Wang M, Lotan R and Lippman SM (1999) Retinoid receptor-dependent and - independent effects of N-(4-hydroxyphenyl) retinamide in F9 embryonal carcinoma cells. *Cancer Res* **59**: 14–18

*© 2001 Cancer Research Campaign*

De Palo G, Veronesi U, Camerini T, Formelli F, Mascotti G, Boni C, Fosser V, Del Vecchio M, Campa T, Costa A and Marubini E (1995) Can fenretinide protect women against ovarian cancer? *J Natl Cancer Inst* **87**: 146–147

Dolo V, Ginestra A, Violini S, Miotti S, Festuccia C, Miceli D, Migliavacca M, Rinaudo C, Romano FM, Brisdelli F, Canevari S, Pavan A and Vittorelli ML (1997) Ultrastructural and phenotypic characterization of CABA I, a new human ovarian carcinoma cell line. *Oncol Res* **9**: 129–138

Fanjul A, Delia D, Pierotti MA, Rideout D, Qiu J and Pfahl M (1996) 4-hydroxyphenyl retinamide is a highly selective activator of retinoid receptors. *J Biol Chem* **271**: 22441–22446

Formelli F, Clerici M, Campa T, Di Mauro MG, Magni A, Mascotti G, Moglia D, De Palo G, Costa A and Veronesi U (1993) Five year administration of fenretinide: pharmacokinetics and effects on plasma retinol concentrations. *J Clin Oncol* **11**: 2036–2042

Formelli F, Barua AB and Olson JA (1996) Bioactivities of N-(4-hydroxyphenyl)retinamide and retinoyl β-glucuronide. *FASEB J* **10**: 1014–1024

Houle B, Rochette-Egly C and Bradley WEC (1993) Tumour-suppressive effect of the retinoic acid receptor β in human epidermoid lung cancer cells. *Proc Natl Acad Sci* **90**: 985–989

Oridate N, Suzuki S, Higuchi M, Mitchell MF, Hong WK and Lotan R (1997) Involvement of reactive oxygen species in N-(4-hydroxyphenyl)retinamide-induced apoptosis in cervical carcinoma cells. *J Natl Cancer Inst* **89**: 1191–1198

Pellegrini R, Mariotti A, Tagliabue E, Bressan R, Bunone G, Coradini D, Della Valle G, Formelli F, Cleris L, Radice P, Pierotti MA, Colnaghi MI and Menard S (1995) Modulation of markers associated with tumor aggressiveness in human breast cancer cell lines by N-(4-hydroxyphenyl)retinamide. *Cell Growth and Diff* **6**: 863–869

Pergolizzi R, Appierto V, Crosti M, Cavadini E, Cleris L, Guffanti A and Formelli F (1999) Role of retinoic acid receptor overexpression in sensitivity to fenretinide and tumorigenicity of human ovarian carcinoma cells. *Int J Cancer* **81**: 829–834

Ponzanelli I, Gianni M, Giavazzi R, Garofalo, Nicoletti I, Reichert U, Erba E, Rambaldi A, Terao M and Garattini E (2000) Isolation and characterization of an acute promyelocytic leukemia cell line selectively resistant to the novel antileukemic and apoptogenic retinoid 6-[3-adamantyl-4-hydroxyphenyl]-2-naphthalene carboxylic acid. *Blood* **95**: 2672–2682

Rozzo C, Chiesa V, Caridi G, Pagnan G and Ponzoni M (1997) Induction of apoptosis in human neuroblastoma cells by abrogation of integrin-mediated cell adhesion. *Int J Cancer* **70**: 688–698

Supino R, Crosti M, Clerici M, Walters A, Cleris L, Zunino F and Formelli F (1996) Induction of apoptosis by fenretinide (4HPR) in human ovarian carcinoma cells and its association with retinoic acid receptor expression. *Int J Cancer* **65**: 491–497

U.K. Coordinating Committee on Cancer Research (1988) UKCCCR guidelines for the welfare of animals in experimental neoplasia. *Br J Cancer* **58**: 109–113

Van den Brule FA, Castronovo V, Menard S, Giavazzi R, Marzola M, Belotti D and Taraboletti G (1996) Expression of the 67 kD laminin receptor in human ovarian carcinomas as defined by a monoclonal antibody, MLuC5. *Eur J Cancer* **32A**: 1598–1602

Van der Leede B-JM, Van den Brink CE, Pijnappel WWM, Sonneveld E, Van der Saag PT, and Van der Burg B (1997) Autoinduction of retinoic acid metabolism to polar derivatives with decreased biological activity in retinoic acid-sensitive, but not in retinoic acid-resistant human breast cancer cells. *J Biol Chem* **272**: 17921–17928

Veronesi U, De Palo G, Marubini E, Costa A, Formelli F, Mariani L, Decensi A, Camerini T, Rosselli del Turco M, Di Mauro MG, Muraca MG, Del Vecchio M, Pinto C, D'Aiuto G, Boni C, Campa T, Magni A, Miceli R and Sporn MB for the Fenretinide Trial Investigators (1999) A randomized trial of fenretinide, a vitamin A analog, to prevent second breast malignancy in women with early breast cancer. *J Natl Cancer Inst* **91**: 1847–1856

 *© 2001 Cancer Research Campaign*
